# Drug Targets for Oxidative Podocyte Injury in Diabetic Nephropathy

**DOI:** 10.7759/cureus.393

**Published:** 2015-12-03

**Authors:** Adnan Bashir Bhatti, Muhammad Usman

**Affiliations:** 1 Department of Medicine, Capital Development Authority Hospital, Islamabad, Pakistan; 2 Department of Medicine, Jinnah Hospital Lahore (JHL)/Allama Iqbal Medical College (AIMC), Lahore, Pakistan

**Keywords:** diabetes, diabetic nephropathy, oxidative podocyte injury, oxidative stress, hyperglycemia

## Abstract

Diabetic nephropathy (DN) is one the most prevalent chronic complications of diabetes mellitus that affects as much as one-third of diabetic patients irrespective of the type of diabetes. Hyperglycemia is the key trigger for DN that initiates a number of microscopic and ultramicroscopic changes in kidney architecture. Microscopic changes include thickening of the glomerular basement membrane (GBM), tubular basement membrane (TBM), mesangial proliferation, arteriosclerosis, and glomerulotubular junction abnormalities (GTJA). Among the ultramicroscopic changes, effacement of podocytes and decrease in their density seem to be the centerpiece of DN pathogenesis. These changes in kidney architecture then produce functional deficits, such as microalbuminuria and decreased glomerular filtration rate (GFR). Among several mechanisms involved in inflicting damage to podocytes, injuries sustained by increased oxidative stress turns out to be the most important mechanism. Different variables that are included in increased production of reactive oxygen species (ROS) include a hyperglycemia-induced reduction in glutathione (GSH), nicotinamide adenine dinucleotide phosphate (NADPH) oxidase activation via hyperglycemia, advanced glycation end products (AGEs), protein kinase C (PKC), and renin-angiotensin-aldosterone system (RAAS).

Unfortunately, control of podocyte injury hasn’t received much attention as a treatment approach for DN. Therefore, this review article is mainly concerned with the exploration of various treatment options that might help in decreasing the podocyte injury, mainly by reducing the level of NADPH oxidase-mediated generation of ROS. This article concludes with a view that certain NADPH oxidase inhibitors, RAAS inhibitors, statins, antidiabetic drugs, and antioxidant vitamins might be useful in decreasing podocyte injury and resultant structural and functional kidney impairments in DN.

## Introduction and background

**Figure 1:**
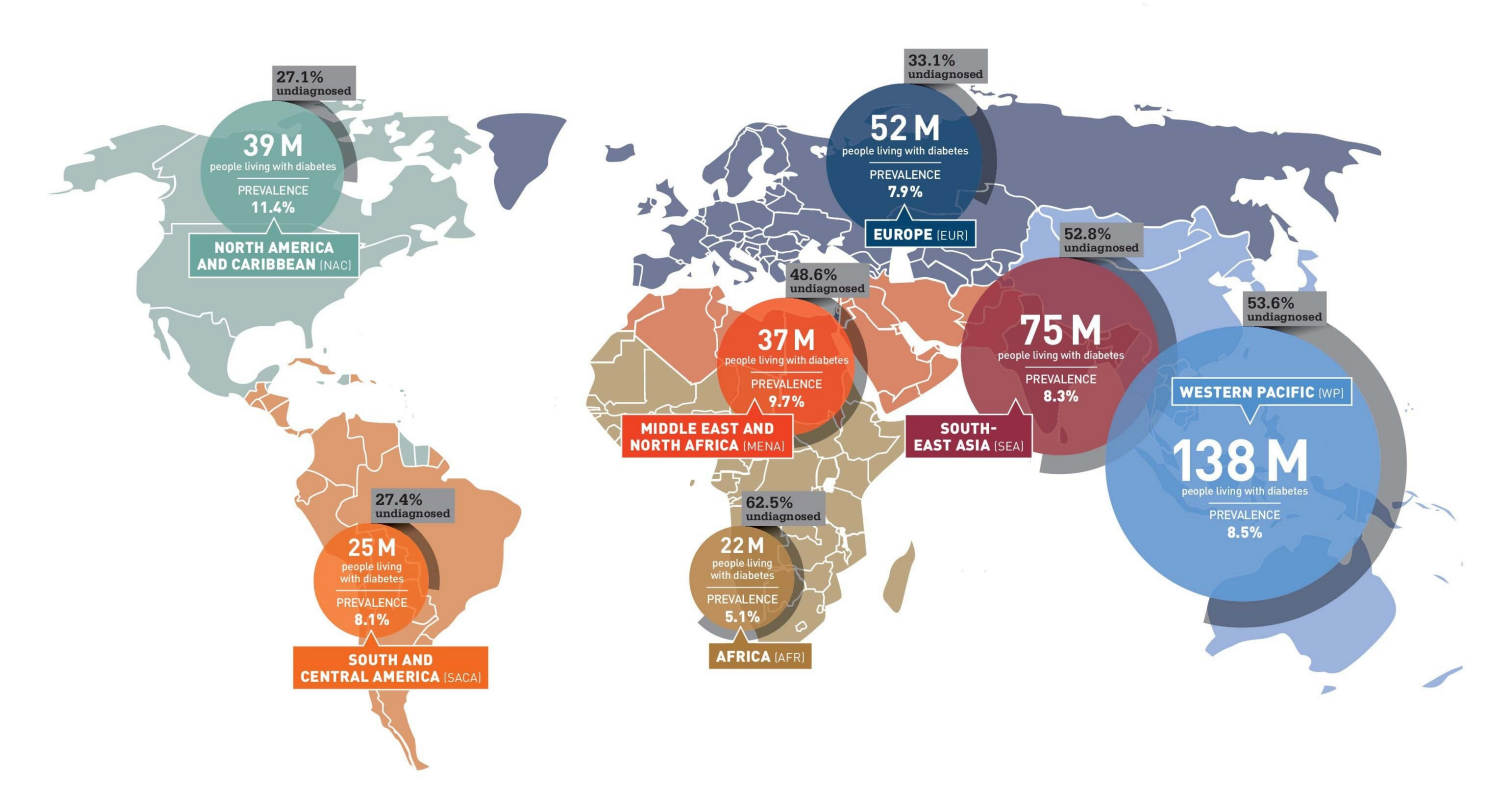
Prevalence of Diabetes. Figure adapted from the diabetes atlas published by International Diabetes Federation (IDF). Retrieved from IDF website on November 12, 2015: http://www.idf.org/sites/default/files/Atlas-poster-2014_EN.pdf

Diabetes is a group of metabolic disorders that is characterized by persistent hyperglycemia either due to the destruction of beta pancreatic cells resulting in a deficit in insulin production or decreased responsiveness of body tissues to secreted insulin (or decreased insulin sensitivity), or both [1-2]. As per 2014 estimates, the global prevalence of diabetes was 8.3%, affecting more than 387 million adults worldwide. These figures are expected to rise to as much as 55% by the end of the year 2030, which will affect more than 592 million adults [3]. The following figure depicts the latest statistics of diabetics worldwide (Figure 1).

Poor glycemic control in diabetes not only increases the risk of acute complications, like hypoglycemia and hyperglycemia [4], but is also responsible for longstanding (chronic) diabetic complications [5]. Long-term complications of diabetes include diabetic nephropathy, peripheral diabetic neuropathy, diabetic retinopathy, autonomic diabetic neuropathy, and cardiovascular complications, such as heart attack and stroke [1, 5-8]. Among these chronic complications, diabetic nephropathy (DN) seems to be the most prevalent as it affects as much as one-third of diabetics irrespective of the type of diabetes they suffer from [9-10]. DN can have fatal consequences that are mostly secondary to kidney failure and cardiovascular complications [11-12]. Therefore, knowing different aspects of DN progression and drug targets can help improve morbidity and mortality status in such patients.

The gross microscopic picture of DN is characterized by increased thickening of glomerular basement membrane (GBM), which is perhaps the earliest detectable lesion in DN [13-14]. As the disease progresses, the thickening of the tubular basement membrane (TBM) soon follows GBM thickening [15]. Thereafter, different degrees of mesangial expansion, which is mainly due to the increased deposition of mesangial matrix and mesangial cellular proliferation, significantly compromises the surface area of the glomerulus that is available for filtration [13, 16-17]. Hyalinosis of afferent and efferent arterioles develops a few years after the initial onset of the disease [18]. Various degrees of glomerulotubular junction abnormalities (GTJA), such as adhesions and obstruction of proximal convoluted tubules, are seen in the later stages of the disease [18-19]. The eventual outcomes of DN include significant atrophy of tubules, focal or segmental glomerulosclerosis, expansion of mesangium and GTJA, which then leads to functional abnormalities like a significant reduction in glomerular filtration rate (GFR) and proteinuria [20-21].

DN in humans is characterized by microalbuminuria, which eventually can progress to proteinuria [22]. Changes in GBM represent an important cause of microalbuminuria; however, a detailed analysis of renal biopsies from patients with Type I and Type II diabetes has demonstrated damage to the cellular elements of the renal glomeruli, which includes visceral epithelial cells and podocytes, as important predictors of functional abnormalities in DN [23-24]. Damage to podocytes represents a significant yet undermined pathological lesion of DN. Analysis of kidney specimens from biopsies of patients with diabetes has shown a marked reduction in the density of podocytes, which was not only evident in patients with advanced disease but was also present in patients with short-term diabetes without microalbuminuria [25-26]. Moreover, experimental models of Type I and Type II diabetes have proved the relevance of podocyte injury in diabetes where depletion of podocytes was found to be the earliest cellular anomaly in DN [27-28]. The relevance of podocyte injury with DN is well-established [29], but what causes this injury is still not completely understood and is a matter of ongoing research.

**Figure 2:**
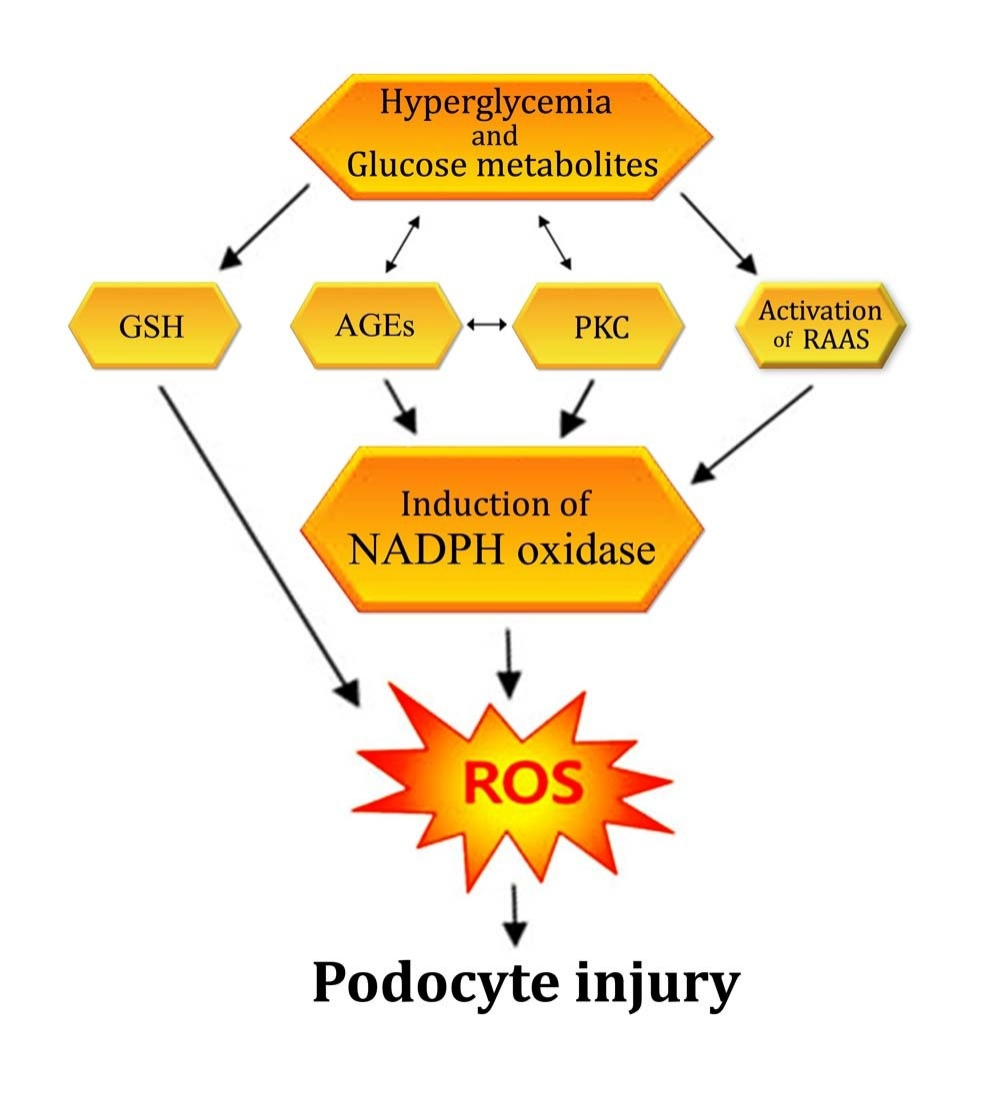
Mechanism of increase in oxidative stress and resultant podocyte injury due to hyperglycemia. High glucose gets metabolized to form obnoxious glucose metabolites; directly reduces the quantity of antioxidants like glutathione (GSH); fuses with proteins to form advanced glycation end products (AGEs); induces protein kinase C (PKC) signaling and deranges renin-angiotensin-aldosterone system (RAAS). All these mechanism tend to increase oxidative stress by inducing the action of NADPH oxidase. The reactive oxygen species (ROS), thus produced, inflict injury to podocytes and decrease their density.

Hyperglycemia seems to be the centerpiece for podocyte injury and DN as a whole, which is most likely due to a higher degree of oxidative stress. Excess glucose is metabolized through multiple accessory pathways like the polyol pathway that converts glucose into sorbitol, which then depletes the amount of antioxidants like glutathione (GSH), and increases the level of reactive oxygen species (ROS) [30]. In addition, increased blood glucose undergoes condensation with free amino acids to form advanced glycation end products (AGEs), which then modulates several important events like the induction of protein kinase C (PKC) [31] and generation of ROS via AGE/a receptor for AGE (RAGE) axis activated nicotinamide adenine dinucleotide phosphate (NADPH) oxidase [32]. Among kinase signaling pathways, the most commonly activated pathway includes the PKC pathway [33], which then triggers the production of ROS via NADPH oxidase [34]. All these pathways are somewhat interlinked, i.e. AGEs and PKC increase the oxidative stress and oxidative stress in turn exacerbates the generation of AGEs and PKC [35-37] (Figure 2). 

A significantly important limb responsible for oxidative stress-induced podocyte damage is the renin-angiotensin-aldosterone axis. Hyperglycemia causes a local increase in blood pressure due to the activation of the renin-angiotensin system (RAS) [38]. In addition, podocytes express functional renin-angiotensin-aldosterone system (RAAS), which includes the angiotensin-converting enzyme (ACE), prorenin receptors (PRR), mineralocorticoid receptors (MR), neprilysin, aminopeptidase A, and renin [39-40]. Studies have demonstrated that activation of angiotensin type 1 receptors (AT1R) by angiotensin II leads to agglomeration of oxidative stress in podocytes during DN [41]. The basic culprit behind RAAS-induced podocyte injury, as shown by studies, is NADPH oxidase [42].

So the question is how the generation of ROS causes damage to podocytes; ROS are well known for their potential to induce apoptosis in cells [43]. Normally, a small amount of ROS is rather essential in maintaining normal cell hemostasis, but hyperglycemia induces production of excessive ROS that results in tissue damage [36]. NADPH oxidase-mediated ROS rise seems to play a fundamental role in podocyte injury in DN. It was demonstrated that in cultured human podocytes, ROS was primarily generated by the NADPH oxidase-mediated pathway, and the podocytes additionally showed the overexpression of NADPH oxidase subunits [44]. The oxidative stress, which is the consequence of the overactivity of NADPH oxidase, thereafter causing cell death due to the triggering of a number of abnormal cell processes. These processes primarily include the damage to the cell DNA, activation of mitochondrial pro-apoptotic factors, and hoarding of aberrant proteins [45-46].

In a crux, it is evident from the above discussion that hyperglycemia-induced oxidative stress is the key event in the injury inflicted to podocytes in DN. Therefore, improvement in our understanding of oxidative stress may prove to be the missing key in the pathogenesis of DN. Moreover, drug targets that can help improve the degree of podocyte survival by decreasing oxidative stress can prove to be valuable assets in treating diabetic patients with DN. Therefore, the purpose of this article is to find possible drug targets that have been shown to decrease ROS-induced podocyte injury and possibly improve DN status in patients suffering from diabetes mellitus. 

## Review

Podocyte injury has been implicated as one of the earliest events in DN. Injury to podocytes due to uncontrolled hyperglycemia and resultant podocyte apoptosis are the most significant events that seem to contribute in decreasing podocyte density, which can be evaluated as microalbuminuria [47]. Diabetes hyperglycemia induces the production of ROS via several mechanisms. NADPH oxidase activation seems to have a pivotal role in all these mechanisms. All of these events then lead to the development of several DN manifestations, including increased podocyte injury [48-50]. Functional loss of kidney structure precedes the loss of podocytes. Therefore, this section is mainly concerned with exploring various therapeutic agents and drug targets that may offer a certain degree of protection to podocytes by decreasing oxidase stress, which may eventually be helpful in treating DN, does not respond well to current treatment options, and has a high morbidity and mortality. The following table summarizes the list of various renoprotective drugs that uphold podocyte architecture (Table 1).

**Table 1:** Overview of renoprotective drugs that uphold podocyte architecture mainly by decreasing oxidative stress.

Group	Drug Name	References
NADPH Oxidases Inhibitors	Triptolide	[52]
	Apocynin	[57]
	Diphenyleneiodonium (DPI)	[59]
RAAS Inhibitors	Enalapril	[66]
	Spironolactone	[69]
	Losartan	[70]
	ACEIs and MR blockers	[71]
Statins	Rosuvastatin	[77]
	Pitavastatin	[78]
Antidiabetic Drugs	Rapamycin	[81]
	Metformin	[84]
Antioxidant Vitamins	Vitamin D	[93]

### NADPH oxidase inhibitors

As discussed above, almost all hyperglycemia-activated pathways induce the production of ROS via the activation of NADPH oxidase. Thus, it can be safely speculated that inhibition of this enzyme may be the most effective therapy in reducing hyperglycemia-induced oxidative stress and resultant podocyte injury. In fact, this hypothesis has been backed by several studies where the use of different oxidase inhibitors has shown significant promise in putting a stop and rather reversing the oxidative stress-mediated podocyte injury. Triptolide is a known anti-inflammatory substance and is well-known for its ability to decrease oxidative stress. It brings about its action by causing inhibition of NADPH oxidase and inducible nitric oxide synthase (iNOS) [51]. Treatment or pretreatment of a rat model of DN with triptolide decreased the production of ROS by the deactivation of NADPH oxidase. The kidney functions improved as shown by reduced proteinuria. Use of triptolide actually reversed the structural damage, as observed as the decrease in the effacement of podocyte foot processes [52]. Apocynin, a potent antioxidant [53], seems to help with podocyte injury through several mechanisms. First, it inhibits the action of NADPH oxidase [54-56]. Second, it mitigates the level of oxidative stress by improving the status of antioxidants like renal glutathione [57]. Diphenyleneiodonium (DPI) brings about its antioxidant effect by causing the inhibition of NADPH oxidase [58]. This activity of DPI also protects podocytes from the damage inflicted by NADPH oxidase-generated ROS [59]. In addition, certain substances, such as triazolopyrimidine, GKT136901, and pyrazolopyridine dione derivatives, have been shown to have a variable degree of NADPH oxidase inhibiting activity, but their role in protecting podocyte injury against oxidative stress and improving the structural and functional impairments in DN has not been extensively studied thus far [60-62].

### Renin-angiotensin-aldosterone system inhibitors

Angiotensin-converting enzyme inhibitors (ACEIs) and angiotensin receptor inhibitors (ARIs) have long been used for the treatment of both non-diabetic and diabetic kidney diseases. How these drugs help protect patients with kidney disease is still not completely understood and cannot be explained by mere regulation of blood pressure [63-64]. Enalapril is an orally prescribed ACEI [65]. Its use has shown to offer a great degree of renoprotective effects in DN. Researchers have demonstrated the antioxidant activity of enalapril in animal models of DN. The study included 24 rats that were divided into three groups: the first group received streptozotocin, the second group received streptozotocin and enalapril, and the third group received placebo (saline only). After seven months of following the protocol, the rats receiving streptozotocin and enalapril showed improvement in kidney structure and function. Researchers concluded that this effect was largely due to the antioxidant activity of enalapril, which includes its ability to improve glutathione status in the kidney [66]. Spironolactone is a known aldosterone receptor antagonist that has been shown to have antioxidant potential. The antioxidant potential of spironolactone can mainly be attributed to its ability to inhibit NADPH oxidase [67]. In addition, it also up-regulates the amount of antioxidants like superoxide dismutase [68]. Researchers have determined the effects of spironolactone administration in animal models of diabetic nephropathy. They concluded that treatment of diabetic rats with spironolactone inhibited NADPH oxidase activity. Moreover, the resultant podocyte injury also diminished following the reduction in oxidative stress [69]. Another RAAS inhibitor is losartan and its metabolites. Recent studies have shown that the metabolites of losartan, EXP3179, but not losartan itself, downregulate the NADPH oxidase activity and NADPH-mediated production of ROS [70]. This property of losartan may also find a way in the treatment of podocyte injury in DN. Moreover, using a combination of RAAS inhibitor drugs may also be helpful in offering a greater degree of renoprotection in subjects with DN. It was also been demonstrated that using a combination of ACEIs and aldosterone receptor blockers (ARBs) offers a greater degree of renoprotection in patients with diabetic nephropathy by the additive inhibition of NADPH oxidase [71].

### Statins

Statins are antihypercholesterolemic drugs that have beneficial antioxidant activity as well. The antioxidant ability of statins can be attributed to two of their properties. First, they decrease the activity of ROS-producing enzymes, such as NADPH oxidase [72-75]. Second, they also increase the level of antioxidants like catalase [76] and superoxide dismutase [77]. Researchers have demonstrated the effects of rosuvastatin administration on an animal model of diabetic nephropathy. The Zucker obese rats were assessed for different variables like the level of oxidative markers (NADPH oxidase), markers of podocyte injury like the effacement of podocyte foot processes, and loss of kidney function as shown by albuminuria. Results showed that treatment of Zucker obese rats with rosuvastatin improved albuminuria, improved the integrity of podocyte filtration barrier, and reduced NADPH oxidase activity [77]. A group of workers have demonstrated the benefits of pitavastatin administration in Dahl salt-sensitive rats. Pitavastatin showed promising results as it protected podocyte injury, mainly by inhibiting the angiotensin type I receptor (AT1R)-induced NADPH oxidase activity [78].

### Antidiabetic drugs

Preferring some antidiabetic drugs over the others can be an interesting and useful treatment option for DN. This treatment option can have two-fold benefits as it only not provides better glycemic control but also provides renoprotection against oxidative stress-induced damage. The first antidiabetic drug that deserves to be mentioned is rapamycin. It has been shown to decrease oxidative stress and improve the function of antioxidant enzymes, such as glutathione [79-80]. Researchers have evaluated the effects of rapamycin administration on rats with experimentally induced DN. Results showed that administration of rapamycin reduced the activity of NADPH oxidase and improved the density of podocytes by inhibiting their apoptosis [81]. Another drug in the antidiabetic category that might make a good addition for the treatment of DN is metformin. The antioxidant potential of metformin is multidimensional. It reduces oxidative stress [82], up-regulates the level of glutathione [83], and suppresses the activity of NADPH oxidase [84]. Results from several studies have persistently supported the benefit of metformin in DN. Metformin decreases oxidative stress through several mechanisms, mainly through the suppression of NADPH oxidase, and by improving the density and functioning of podocytes [85-88].

### Antioxidant vitamins

Among vitamins, the vitamins that might be useful in the treatment of DN include antioxidant vitamins like vitamins C, D, and E. Vitamin D is a well-known antioxidant [89] and inhibitor of NADPH oxidase [90]. Several studies have shown the efficacy of vitamin D in saving podocytes from oxidative stress, mainly due to its antioxidant action [91-93]. Other antioxidant vitamins that might help uplift the health of podocytes include alpha-tocopherol and ascorbic acid. It has been demonstrated that co-administration of puromycin, a known reno-toxic drug, and alpha-tocopherol/ascorbic acid can help decrease podocyte damage and increase podocyte density [94]. The efficacy of these vitamins in relation to DN has not yet been elucidated. The evaluation of benefits of these antioxidant vitamins in DN demands further research. 

## Conclusions

Podocyte injury represents a centripetal event in the progression of DN and related functional deficits like microalbuminuria and decreased GFR. NAPDH oxidase-mediated oxidative stress seems to be the most important event in the oxidative podocyte injury. Increased production of ROS then inflicts serious damage to the podocytes and leads to slowly progressing deterioration in kidney structure and function. Thus, blocking the production of ROS can be a valuable treatment approach for decreasing podocyte injury and resultant kidney disease. In the light of current literature, we have concluded that certain NADPH oxidase inhibitors, RAAS inhibitors, statins, antidiabetic drugs, and antioxidant vitamins have shown significant potential in decreasing oxidation-induced podocyte injury. These therapeutic agents have shown positive results in animal models of DN, but clinical trials to see their effects on humans are rather lacking. There is a need for research and clinical trials using these therapeutic agents to study their efficacy for managing DN.
